# Spire, an Actin Nucleation Factor, Regulates Cell Division during *Drosophila* Heart Development

**DOI:** 10.1371/journal.pone.0030565

**Published:** 2012-01-20

**Authors:** Peng Xu, Tamara L. Johnson, Jessica R. Stoller-Conrad, Robert A. Schulz

**Affiliations:** Department of Biological Sciences, University of Notre Dame, Notre Dame, Indiana, United States of America; University of Massachusetts Medical School, United States of America

## Abstract

The *Drosophila* dorsal vessel is a beneficial model system for studying the regulation of early heart development. Spire (Spir), an actin-nucleation factor, regulates actin dynamics in many developmental processes, such as cell shape determination, intracellular transport, and locomotion. Through protein expression pattern analysis, we demonstrate that the absence of *spir* function affects cell division in Myocyte enhancer factor 2-, Tinman (Tin)-, Even-skipped- and Seven up (Svp)-positive heart cells. In addition, genetic interaction analysis shows that *spir* functionally interacts with *Dorsocross*, *tin*, and *pannier* to properly specify the cardiac fate. Furthermore, through visualization of double heterozygous embryos, we determines that *spir* cooperates with *CycA* for heart cell specification and division. Finally, when comparing the *spir* mutant phenotype with that of a *CycA* mutant, the results suggest that most Svp-positive progenitors in *spir* mutant embryos cannot undergo full cell division at cell cycle 15, and that Tin-positive progenitors are arrested at cell cycle 16 as double-nucleated cells. We conclude that Spir plays a crucial role in controlling dorsal vessel formation and has a function in cell division during heart tube morphogenesis.

## Introduction

The *Drosophila* heart, also called the dorsal vessel, is a simple organ that acts as a myogenic pump to allow the circulation of hemolymph throughout the body. It is a proven model for studying cell-cell signaling and the regulation of early heart development. The dorsal vessel consists of 104 cardioblasts, which can be individually identified *in vivo*, such that differentiation and cell fate analysis can be conducted at the resolution of a single cell and followed throughout the whole developmental process [1]. Most segments of the dorsal vessel contain six pairs of cardioblasts: four pairs of Tinman (Tin)-positive cells that have large nuclei and two pairs of Seven up (Svp)-positive cells with smaller nuclei [2].

In the cardiogenic mesoderm, three transcriptional effectors, Tin, Pannier (Pnr) and Dorsocross (Doc), are essential for the generation of cardiac progenitors and cardioblast specification [3]. Mutations in any one of these genes cause severe defects in dorsal vessel formation. These three genes are activated by Decapentaplegic and Wingless signaling pathways and regulate each other. Subsequently these transcription factors, individually or in combination, activate known and yet to be discovered target genes that lead to the appropriate morphological structures of the dorsal vessel, as well as the proper differentiation of cardioblasts [2].

Cardiac progenitors arise from a discrete cell population that is generated during early embryonic mitosis. Three embryonic divisions (mitosis 14–16) are subsequent to interphase 14 in most cell lineages. The third division (mitosis 16) takes place during late stage 10 to stage 11 between 280–300 minutes after egg deposition. This division is characterized by a continuous longitudinal region that generates heart precursors and the development of a portion of the visceral mesoderm [4]. Cyclin A (CycA), which is the only cyclin necessary for mitosis in *Drosophila*, is found in the cytoplasm in interphase and accumulates in the nucleus during prophase [5], [6]. In addition, it was previously shown that CycA can compensate for Cyclin B in mitotic entry [7]. The last round of global cell division, mitosis 16, arrests in *CycA* mutants [7], [8].

Recently, several screens have been undertaken to identify new genes involved in cardiogenesis [9]. Through the use of a *Df(3L)DocA* mutant allele, previous studies have demonstrated that *Doc* interacts with *tin*, *pnr* and *tailup* (*tup*) [3], [10]. We completed a sensitized screen, using *Df(3L)DocA* as the background, to find genes that could potentially interact with *Doc* in heart development. Our screen identified *spire* (*spir*) as another *Doc*-interacting gene.

Spir is an actin-nucleation factor, which regulates actin dynamics in developmental processes such as cell shape determination, intracellular transport, and division [11], [12]. Spir protein orthologs from different species share a common structural array: a KIND domain at the N-terminus, a cluster of four Wiskott-Aldrich syndrome protein-homology domain 2 (WH2) domains in the central region, and a Spir-Box and a FYVE membrane-binding domain at C-terminus [12]. The highly conserved WH2 domains are necessary and sufficient for the actin-nucleation activity of the protein [11].

Previous studies of Spir have focused on its function during *Drosophila* oogenesis, especially in domain interactions with Cappuccino [13]–[15]. In our current study, we demonstrate that Spir is ubiquitously expressed during embryogenesis and that cell division of Myocyte enhancer factor 2 (Mef2)-, Tin-, Even-skipped (Eve)- and Svp-positive heart cells are affected in the absence of *spir*. Furthermore through genetic analyses, we find that *spir* functionally interacts with *Doc*, *tin, pnr* and *CycA* to regulate cardiac fate and cell division. In addition, our results suggest that in *spir* mutant embryos, most Svp-positive progenitors fail to undergo full cell division at cell cycle 15. Tin-positive progenitors cannot divide at cycle 16 resulting in double-nucleated cells. Based on these findings, we believe that Spir plays a crucial role in cell division during heart tube morphogenesis and dorsal vessel formation.

## Results

### Spatial and temporal expression of Spir during embryogenesis

In *Drosophila*, several different Spir isoforms are encoded by the *spir* locus, three of which are well characterized: 1) the full-length Spir transcript, SpirA; 2) a splice variant that includes KIND and WH2 domain cluster, SpirD; and 3) a splice variant that includes the C-terminal region encoding the Spir-box and the FYVE zinc finger, SpirC.

**Figure 1:**
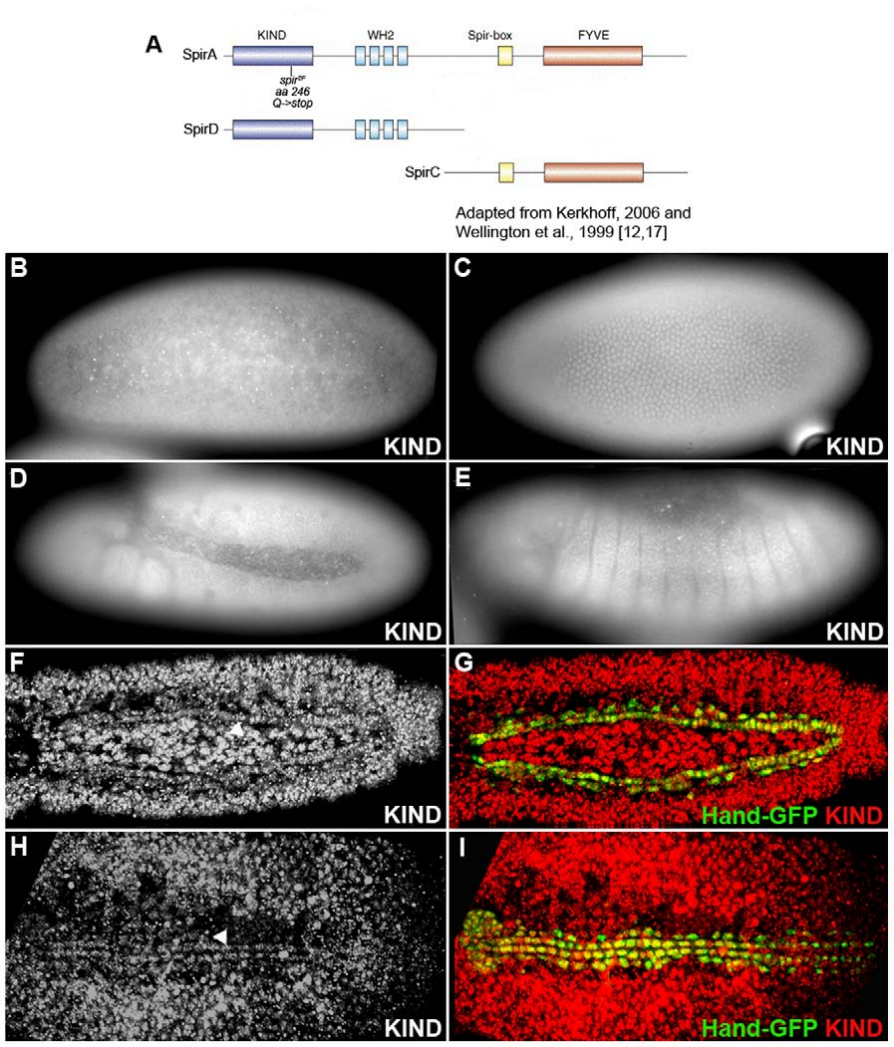
Spir expression pattern during *Drosophila* embryogenesis. (A) Diagram of SpirA, SpirC and SpirD proteins and the location of *spir^2F^* mutant. Anti-KIND antibody recognizes KIND domain in both SpirA and SpirD proteins. Anti-SpirD antibody recognizes SpirD and the N-terminus of the A-isoform. (B–I) Whole-mount antibody stainings of wild-type embryos with anti-KIND. Anti-SpirD antibody exhibits same staining pattern (data not shown). (B) Stage 4. (C) Stage 5. Focus on the lateral epidermis. (D) Stage 11. Fully extended germ band. (E) Stage 13 (lateral view). (F, G) Stage 15. Dorsal view to visualize the distinct accumulation of Spir in cardioblasts and pericardial cells. (H, I) Stage 16 (Dorsal view). *Hand-GFP* is expressed in cardioblasts, pericardial cells and the lymph gland. The Spir expression is co-localized with *Hand-GFP* in heart cells. Arrowheads point to cardioblasts.

Spir protein showed a ubiquitous expression pattern during embryogenesis (Fig. 1). Staining by anti-SpirC showed SpirC expressed in nuclei and cytoplasm ubiquitously (data not shown). An anti-KIND antibody recognizes the KIND domain at the N-terminus of the Spir protein and has been shown to accurately label SpirA and SpirD expression during *Drosophila* oogenesis [14]. An anti-SpirD antibody recognizes SpirD and the N-terminus of SpirA [16]. From early embryonic stages through stage 13, SpirA and SpirD were localized in nucleus and cytoplasm of every cell through anti-KIND and anti-SpirD staining (Fig. 1A–D). From stage 14 to 16, SpirA and D localization changed to be expressed in the nucleus of every cell (Fig. 1E, G). It was identified that at these stages when the heart cells were fully differentiated, both the KIND domain and SpirD co-localize with the heart specific cell marker, *Hand-GFP*, in the nuclei of both cardioblasts and pericardial cells (Fig. 1F, H). This indicates that Spir is expressed within the nuclei of heart cells at late embryonic stages.

### Analysis of *spir* mutant phenotypes during heart development

**Figure 2:**
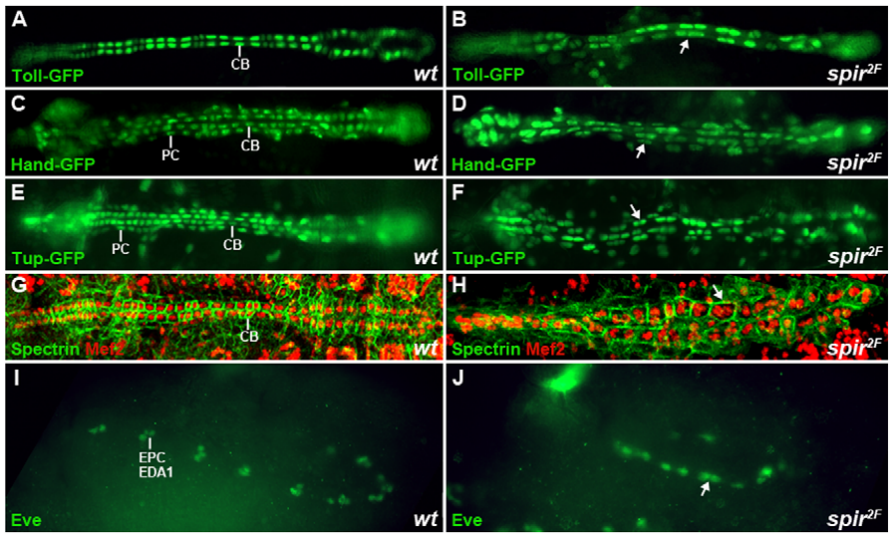
Heart phenotypes in spir^2F^ mutants. (A–H) Compared to wild-type *Drosophila* embryos, *spir^2F^* mutant embryos are characterized by paired nuclei, larger cells and fewer cardioblasts at stage 16. (A, B) The *Toll-GFP* transgene expresses GFP in cardioblasts. (C, D) The *Hand-GFP* transgene and (E, F) the *Tup-GFP* transgene expresses GFP in cardioblasts, pericardial cells and the lymph gland. (G, H) Spectrin (green) and Mef2 (red) stain membrane skeleton and cardioblasts, respectively. (I, J) At stage 12, Eve stains pericardial cells and DA1 cells. In *spir^2F^* mutant embryos, Eve-positive cells appear in pairs. All embryos are oriented with the anterior to the left. All the images are taken under 40× magnification. Arrows indicate paired nuclei in *spir* mutants. Pericardial cells (PC), cardioblasts (CB), Eve-positive pericardial cells (EPC) and Eve-positive DA1 cells (EDA1) of the normal dorsal vessel are indicated in the wild-type embryos.

In order to assess *spir* function during heart development, various heart cell markers were used to analyze heart development in *spir* mutant embryos. In this study, we used a truncation mutant, *spir^2F^*, which has a stop-codon at amino acid 246, such that the protein does not include the four WH2 domains [17]. Initially, we used the transgenic GFP lines *Hand-GFP* and *Tup-GFP*, which mark cardioblasts and pericardial cells, and *Toll-GFP*, which marks cardioblasts and amnioserosa cells, to investigate the *spir* mutant phenotype *in vivo*. This allowed us to easily distinguish cells and count them accurately [18], [19]. In *spir* mutants, the nuclei of heart cells were elongated and slightly larger than those of normal heart cells (Fig. 2B, D, F). Additionally, these nuclei appeared in pairs within single cells as detected by a Spectrin antibody, which marks the membrane skeleton and allows clear visualization of the cell edge [20] (Fig. 2H). The average number of nuclei was 78±6 (n = 20). Based on the Spectrin staining, it was clear that in *spir* mutants, the size of the heart cells was much larger than that of normal heart cells, and that in most cells, there were two nuclei. This incomplete cell division resulted in a cardiac cell number much less than the normal 104 cells [2] (Fig. 2G, H).

**Figure 3:**
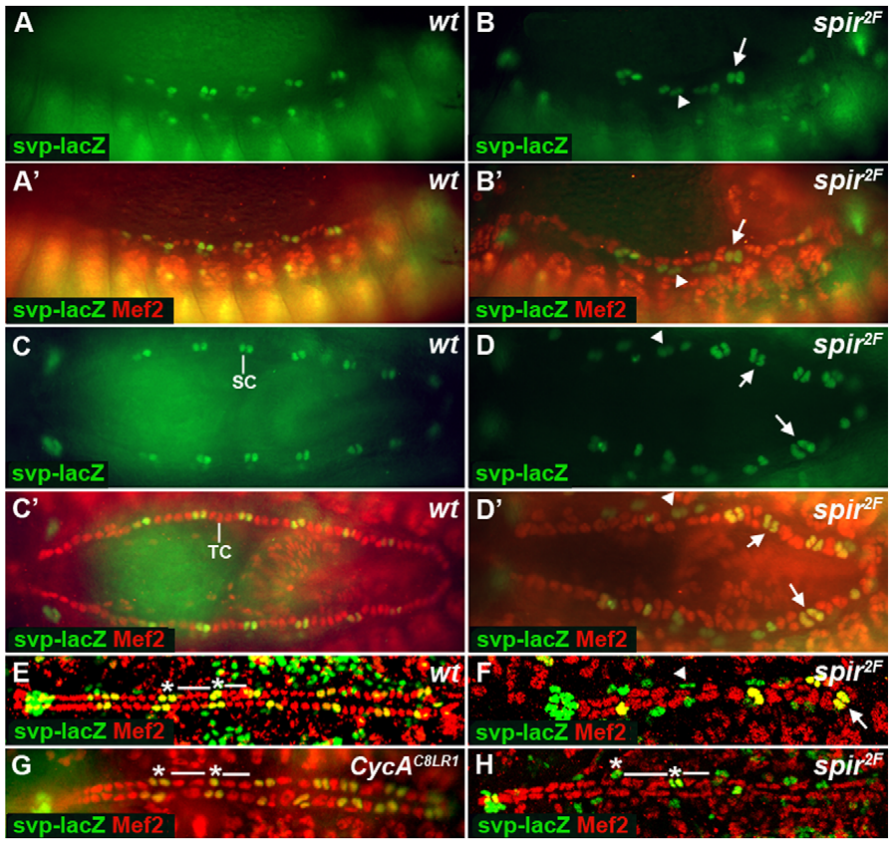
Pattern of Tin- and Svp-positive cells in *wt*, *CycA^C8LR1^* and *spir^2F^* embryos. (A, A′, B and B′) Lateral view at stage 13. (C, C′, D and D′) Dorsal view at stage 15. (E–H) Dorsal view at stage 16. Mef2 (red) stains Mef2-positive cardioblasts and β-Gal (green) stains Svp-positive cardioblasts. In *spir^2F^* mutants, when two nuclei appear together, most cells express only Svp. When three or four nuclei appear together, they are stained with both anti- β-Gal and anti-Mef2. SC, Svp-positive cardioblasts; TC, Tin-positive cardioblasts. Arrows point to Svp-positive cardioblasts; Arrowheads point to Svp-positive pericardial cells. * indicates Svp-positive cells; — indicates Tin-positive cells.

To determine if *spir* affected early heart development, embryos were stained with anti-Eve antibody. In stage 12 wild-type embryos, Eve progenitor cells differentiate into the Eve-positive pericardial cells and DA1 muscle cells of each hemi-segment, which appear in 2–3 cell clusters (Fig. 2I). In *spir* mutant embryos, there were no cell clusters, but the nuclei were in pairs (Fig. 2J). Finally, we analyzed the pattern of Svp- and Tin-positive heart cells by Svp-lacZ and Mef2 staining. The *svp* gene is the *Drosophila* homolog of the vertebrate COUP-TF nuclear transcription factor, a key regulator expressed in the non-Tin-expressing cardioblasts which represses *tin* expression in those cardioblasts [21]. In wild-type animals, Mef2 is expressed in all cardioblasts and within each heart segment there are two pairs of Svp-positive cardioblasts and four pairs of Tin-positive (Fig. 3C′, E). Compared to 14 pairs of Svp-positive cardioblasts in the normal heart, in *spir* mutants there were only an average of 12±4 Svp-positive cardioblast nuclei (n = 20; Fig. 3B′, D′, F, H). Together, these results suggest an important role for *spir* in proper cell division and specification including Mef2-, Tin-, Svp-, Eve-, *Hand-* and *Tup-*positive heart cells. In spir mutants, the division of the muscle cells were also abnormal as marked by Mef2 antibody and *Tup-GFP* indicating that it possibly functions widely in cell division during embryogenesis. However, this study specifically focused on *spir* function in heart development.

In order to study which stage of the cell cycle was affected by the *spir* mutation, we compared the expression pattern of Svp and Tin in *spir* mutants to that of *CycA* mutants, which causes cell cycle arrest at mitosis 16 [7]. It has been found that in *CycA* mutants, the number of Mef2-positive cardioblasts is reduced from six to four per hemi-segment, consisting of two Tin-positive cells and two Svp-positive cells, with one nucleus per cell (Fig. 3G) [22]. In contrast to wild-type and *CycA* mutants, *spir* mutants showed that in cells with 3 or 4 nuclei together, the nuclei were stained by both anti-Svp and anti-Mef2 antibodies, which suggested that they were Svp-positive cardioblasts. However, cells with paired nuclei that expressed only Svp, and not Mef2, were Svp-positive pericardial cells (n>50 *spir* mutant embryos) (Fig. 3B′, D′, F, H). This investigation suggests that Spir affects the fate of Svp-positive progenitors.

### *spir* interacts with *tin*, *pnr* and *Doc* during cardiogenesis

**Figure 4:**
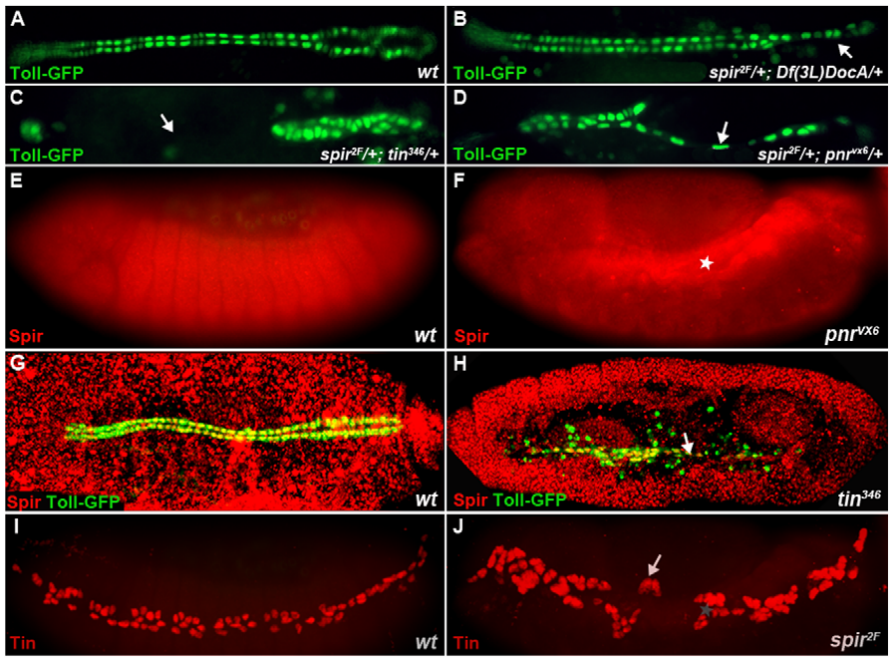
Genetic interactions between *spir* and *Doc*, *tin*, *pnr*. (A) Wild-type embryo. (B–D) Double heterozyous embryos of *spir* and *Df(3L)DocA*, *spir* and *tin* and *spir* and *pnr*, respectively. Embryos are all at stage 16. The mutant phenotypes of all three double heterozygous embryos show missing cardioblasts. (E–H) Spir antibody staining in *wt* and *pnr* mutant, *wt* and *tin* mutant embryos, respectively. (E, F) In *pnr* mutant embryos, Spir is over-expressed in the cardiac mesoderm, compared to the ubiquitous expression pattern in wild-type embryo at stage 13. (G, H) There is no Spir expression in heart cells in *tin* mutants at stage 16. The cells that express *Toll-GFP* and Spir are amnioserosa cells. (I, J) Tin antibody staining in wild-type embryo (I) and *spir* mutant embryo (J) at stage 13. Tin cells appear in pairs and are disorganized. Arrows highlight the abnormal positioning of cardioblasts in different mutant embryos. ★ indicates cardiac mesoderm.

Doc, Tin, and Pnr are essential transcription factors, known to regulate each other and function in the specification of cardioblasts [3]. We tested double heterozygous mutant embryos: *spir^2F^/+; Df(3L)DocA/*+, *spir^2F^/+; tin^346^/+,* and *spir^2F^/+; pnr^VX6^/+*, to determine the interaction between *spir* and each of these factors, respectively. The phenotypes of these double heterozygous embryos were compared with the phenotypes of single heterozygous embryos for each of the investigated alleles. Although most embryos had normal numbers of cardioblasts, a small fraction of single heterozygous *spir^2F^*, *Df(3L)DocA, tin^346^*, and *pnr^VX6^* embryos presented with reduced numbers of cardioblasts, indicating that the decreased activity of these genes inhibited normal cardioblast development (data not shown). However, in all groups, the ratio of the number of mutant hearts to normal hearts was significantly different when comparing single heterozygous and double heterozygous embryos (Table 1). When *spir^2F^* was combined with either *tin^346^* or *pnr^VX6^*, the double heterozygous embryos showed obvious gaps within the *Toll-GFP* myocardial cell rows in ∼20% of the embryos analyzed. When combined with *Df(3L)DocA*, this percentage was approximately 10% (Fig. 4B–D, Table 1). Interestingly, in *spir* and *pnr* double heterozygous embryos, there were elongated nuclei similar to those in *spir* mutants. A Chi-square test showed that the interactions between *spir* and *pnr, spir* and *tin* were stronger than the interaction between *spir* and *Doc*, as indicated by the p value (P_pnr_<0.0001; P_tin_<0.0001; P_Doc_ = 0.06, Table 1). This analysis indicates that *spir* is required in combination with *tin, pnr* and *Doc* to properly specify cardioblasts.

**Table 1:** Statistical analysis of genetic interaction between *spir* and *tin*, *spir* and *Doc*, *spir* and *pnr*, *spir* and *CycA*.

Genotype	Normal hearts	Mutant hearts	%	Genotype	Normal hearts	Mutant hearts	%
*spir^2F^/+; tin^346^/+*	153	43#	22%	*spir^2F^/+; Df(3L)DocA/+*	217	23#	10%
*tin^346^/+*	249	30	11%	*Df(3L)DocA/+*	249	14	5%
*spir^2F^/+*	257	13	5%	*spir^2F^/+*	257	13	5%
P value	P_tin_<0.0001**				P_Doc_ = 0.06*		
*spir^2F^/+; pnr^vx6^/+*	134	41#	23%	*spir^2F^/+; CycA^C8LR1^/+*	76	43	36%
*pnr^vx6^/+*	136	13	9%	*CycA^C8LR1^/+*	297	10	3%
*spir^2F^/+*	257	13	5%	*spir^2F^/+*	257	13	5%
P value	P_pnr_<0.0001**				P_CycA_<0.0001**		

**The χ^2^ test reveals in all three groups that the proportion of mutant and normal embryos is statistically different for single heterozygous and double heterozygous embryos.

*The χ^2^ statistic is not significant at the 0.05 level, but at the 0.1 level.

# Mutant hearts in these double heterozygotes denote gap phenotype.

To further evaluate the functional relationships between *spir* and *tin*, *pnr* and *Doc*, we analyzed the expression of Spir in *Df(3L)DocA*, *pnr^VX6^* and *tin^346^* mutant embryos. Staining of Spir protein in *Df(3L)DocA* embryos showed that Spir was expressed ubiquitously (data not shown). In half of *pnr* mutant embryos, Spir was over-expressed in cardiac mesoderm (Fig. 4F). In *tin^346^* mutant embryos, Spir staining was absent in cardioblasts because as a consequence of the mutation, no heart cells were found (Fig. 4H).

*spir^2F^* mutant embryos were also stained with anti-Tin, anti-Pnr and anti-Doc antibodies. At stage 11, Tin, Pnr and Doc are all expressed in cardiac mesoderm in the *Drosophila* embryo [3]. Later in development, *tin* expression continues in a certain set of cardioblasts and pericardial cells [20], [23], [24]. The expressions of Doc and Pnr cease during stages 12 and 13. However, Doc expression is weakly maintained in Svp-expressing cardioblasts until the end of embryogenesis [21], [25]. At stage 13 in *spir^2F^* mutants, Tin stained nuclei were close together and appeared in pairs. This result reaffirmed previous findings suggesting defects in Tin-positive cardiac precursor cell division (Fig. 4J). However, the Pnr and Doc expression patterns were unchanged in *spir^2F^* mutant embryos at stage 13 (data not shown). Our results, combined with the data above, suggest that *spir* genetically interacts with *tin*, *Doc*, and *pnr* to different degrees to regulate heart cell division.

### *spir* interacts with *CycA* during dorsal vessel formation

**Figure 5:**
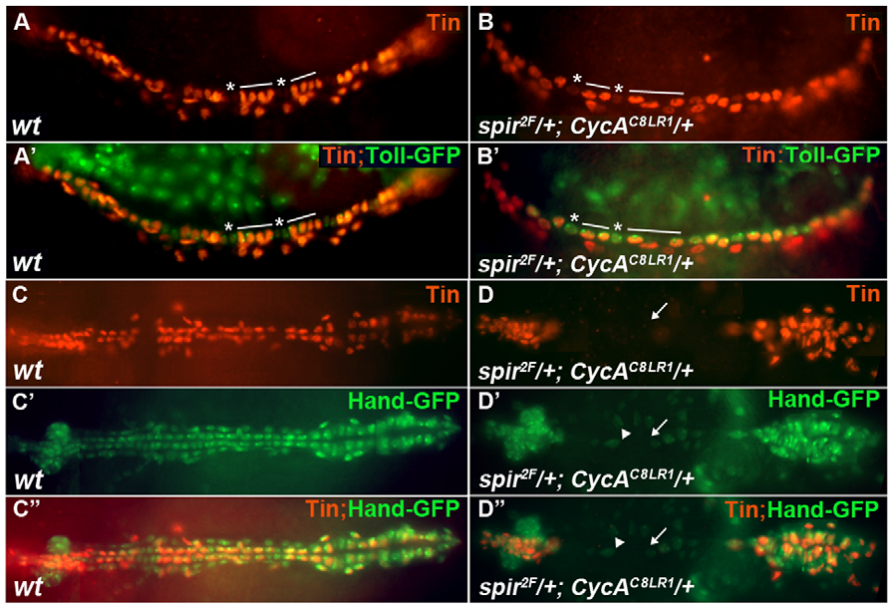
Genetic interaction between *spir* and *CycA*. This interaction results in various phenotypes. (A, A′, B and B′) Stage 13. In each hemi-segment, there are two Tin-positive cells and one Svp-positive cell in *spir* and *CycA* double heterozygous embryos (B and B′). (C–C″ and D–D″) Stage 16. Many cells are missing in the aorta region (D–D″). Many mutant embryos show a phenotype between these two extremes. Arrows emphasize cellular gaps in the dorsal vessel. * indicates Svp-positive cells; — indicates Tin-positive cells. Arrowheads point to paired nuclei. Arrows point to the missing cells.

We next investigated a possible genetic interaction between *CycA* and *spir*. In *CycA* and *spir* double heterozygous embryos, 35% of the embryos showed either abnormal cell division or a significant loss of heart cells, especially in the aorta region of the heart tube (Fig. 5B, D). Some double heterozygous embryos showed that the Tin- and Svp-positive cell divisions were both affected by the double heterozygous mutations. For example, some double heterozygous mutants formed two Tin-positive cells and one Svp-positive cell in each hemi-segment (Fig. 5B, B′). When compared to the *CycA* mutant phenotype, one Svp-positive cell was missing in each hemi-segment of the double heterozygotes. In contrast to the *spir* mutants, there were no paired nuclei in the double heterozygous embryos. This result suggests that *spir* interacts with *CycA* and leads to the abnormal cell division from both Tin- and Svp-positive super-progenitors (preprogenitors) to progenitors (cycle 15) as indicated by our model discussed later. Other double heterozygous embryos showed large gaps formed in the heart tube due to loss of many heart cells, indicating that there were no precursor cells formed which resulted in the absence of cardioblasts at the later stages (Fig. 5D–D″). Chi-square analysis also demonstrated that the combined action of *spir* and *CycA* was required for proper cardiac cell division (P_CycA_<0.0001, Table 1).

## Discussion

Cardiac cell fate specification requires the coordinated function of multiple factors such as *tin*, *pnr*, and *Doc*. *Drosophila* is an ideal model for the determination of the complex transcriptional network that initiates and maintains the cardiac lineage. Our data place the nucleation factor *spir* as an integral factor in cardiac cell division and dorsal vessel formation.

Proper dorsal vessel morphogenesis is critically dependent upon intercellular signaling and the regulation of gene expression. Although great progress has been made in the study of heart development, it is not known exactly how many genes and pathways are involved in this cardiogenic process or how many of these factors cooperate together. Previous genetic screens have identified genes that play roles in the specification, morphogenesis, and differentiation of the heart, including *mastermind* and *tup* [10], [26], [27]. Our sensitized screen has also been proved as an efficient method to find additional factors in this process, suggesting that much remains to be learned about the molecular components involved in correct dorsal vessel formation.

**Figure 6:**
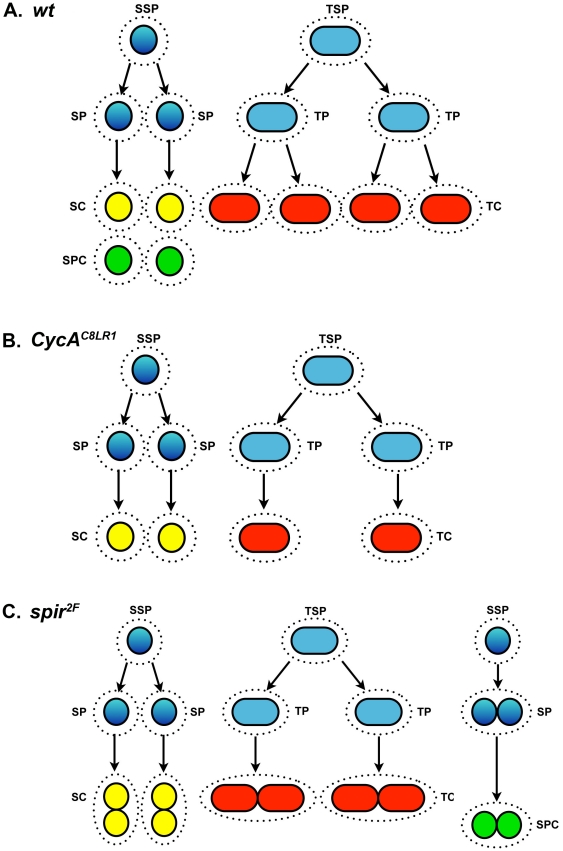
A cell division model of *spir* function during heart development. (A) In wild-type embryos, there are six cardioblasts in each hemi-segment including two Svp-positive and four Tin-positive cells. An SSP divides into two SPs, then each of the SPs divides into one SC and one SPC. After two cell cycles, one TSP differentiates into 4 TCs. (B) In *CycA* mutants, cell cycle 16 is blocked so that there are two SCs and two TCs in each hemi-segment. However, there are no SPCs. (C) In *spir* mutants, the nucleus duplicates and divides while the cytoplasm does not. Most of the SSPs stop division at cell cycle 15 resulting in double-nucleated cells. Some of the SSPs divide into two SPs, but stop at cell cycle 16 giving rise to four or three nuclei clusters. TPs are arrested at cell cycle 16 leading to two double-nucleated cells in each hemi-segment. SSP, Svp-positive super-progenitor; TSP, Tin-positive super-progenitor; SP, Svp-positive progenitor; TP, Tin-positive progenitor; SC, Svp-positive cardioblasts; TC, Tin-positive cardioblast; SPC, Svp-positive pericardial cells. The dotted circle represents the cell membrane.

Spir is required for the proper timing of cytoplasmic streaming in *Drosophila*, and loss of *spir* leads to premature microtubule-dependent fast cytoplasmic streaming during oogenesis, the loss of oocyte polarity, and female sterility [28]. Even though it is known that *spir* is an important actin filament nucleation factor, our findings are the first report to describe a function of *spir* for cell division. Through phenotypic analysis of the *spir* mutant phenotype, we find that many cardioblast nuclei are partially or completely divided. However, the cytoplasm is not divided in the absence of *spir*, which is consistent with the function of *spir* in cytoplasmic movement. Thirteen rapid nuclear division cycles without cell division initiate *Drosophila* embryo development, followed by three waves of cell division [29]–[31]. The first wave of cell division occurs in the mesoderm at cell cycle 14. After this initial division, cells migrate, spread dorsally and undergo a second round of cell division at cell cycle 15. The third wave of cell division in the mesoderm occurs at the end of germband extension during cell cycle 16. There are two different types of cardioblast precursor cells: one type divides into two identical Tin-positive cardioblasts (TC), and the other type divides into one Svp-positive cardioblast (SC) and one Svp-positive pericardial cell (SPC) [24]. Based on the comparison of *CycA* and *spir* mutant phenotypes, we predict a tentative cell division model to demonstrate *spir* function in determining cardiac cell fate (Fig. 6). Han and Bodmer [22] demonstrate that in a wild-type background, one Svp-positive super progenitor (SSP) divides into two Svp-positive progenitors (SP), then each of these cells divides into one SPC and one SC. For Tin-positive super progenitors (TSP), after each divides into two Tin-positive progenitors (TP), each TP further divides into two identical TCs (Fig. 6A). In our model, division from the super progenitor to progenitors takes place at cell cycle 15, and division from progenitors to full differentiated heart cells occurs at cell cycle 16. In *CycA* mutants, mitosis 16 is blocked such that both SPs and TPs stop cell division. This results in the two SPs assuming a myocardial fate. Thus the number of SCs remains normal, but half of the TCs are missing in the *CycA* mutants [22] (Fig. 6B). Our data suggest that in *spir* mutant embryos, most of the SPs fail to undergo full cell division at cycle 15 resulting in a SPC fate with paired nuclei. A subset of these cells are able to undergo the 15th cell division but are arrested at cycle 16 as double-nucleated cells which exhibit both Svp and Mef2 staining, characteristic of the SCs seen in the *CycA* mutants. Similarly, for TPs, cycle 16 was also blocked such that it resulted in two double-nucleated cells. In summary, Spir affects mitosis 16 for Tin-positive cell division and both mitosis 15 and 16 for Svp-positive cell division (Fig. 6C).

Antibody staining implicates that Spir is expressed ubiquitously before stages 12–13 and is located in both nuclei and cytoplasm. After cell cycle 16 cell division stops, occurring during stage 10–11. The expression of Spir in the cytoplasm then decreases gradually. At stage 15, the staining pattern shows mostly nucleus expression with some cytoplasmic expression and by stage 16 the nuclei become distinct indicating nucleus staining only. It is hypothesized that expression of Spir decreases in the cytoplasm but remains constant in the nuclei when cell division halts.

Our genetic analysis of *spir*, *Doc*, *pnr* and *tin* suggests that these factors may regulate each other during dorsal vessel formation, and especially significant is the interaction between *spir* and *pnr*. Pnr is a GATA class transcription factor expressed in both the dorsal ectoderm and dorsal mesoderm, where it is required for cardiac cell specification. Proper dorsal vessel formation is inhibited in *pnr* loss-of-function embryos due to failure in the specification of the cardiac progenitors [32]–[34]. In *spir* mutants, the expression pattern of Pnr remains normal. However, Spir is over expressed in the cardiac mesoderm in *pnr* mutants, suggesting that Pnr may repress the expression of the *spir*.

In conclusion, Spir is a newly-identified factor functioning in cell division during dorsal vessel formation. Tin-, Eve- and Svp-positive heart cells are all affected in the absence of *spir*. Also, *spir* expression depends on the transcription factors *Doc*, *tin* and *pnr*. Genetic interaction data also show that *spir* cooperates with *CycA* in heart cell division.

## Materials and Methods

### *Drosophila* strains and genetics

The following mutant fly stocks were obtained from the Bloomington Stock Center: *spir^2F^*, *spir^1^*, *pnr^VX6^*, *CycA^C8LR1^*, *Svp-lacZ/TM3, w; noc^sco^/CyO, P{Dfd-EYFP}2*, *w; ry^506^ Dr^1^/TM6B, P {Dfd-EYFP} 3, Sb^1^ Tb^1^ ca^1^*, *w; In(2LR)noc^4L^Sco^rv9R^, b^1^/CyO, P{ActGFP}JMR1*. *Df(3L)DocA* [25] and *tin^346^* [35] were obtained from Dr. M. Frasch (Friedrich-Alexander University of Erlangen-Nuremberg, Germany). The *Hand-GFP* transgenic stock was obtained from Dr. Z. Han (University of Michigan, Ann Arbor, MI). The *Toll-GFP* and *Tup-GFP* transgenic reporters were maintained by our lab [18], [27].

The *pnr^VX6^, tin^346^* and *Df(3L)DocA* were rebalanced with *TM6B, P{Dfd-EYFP}3* and combined with *Toll-GFP* on the X chromosome. The *spir^2F^* was rebalanced with *CyO, P{Dfd-EYFP}2*. This *Dfd-EYFP* driven by *deformed* enhancer can score mid- to late-stage embryos, which facilitates phenotype analysis. For the genetic interaction analysis, embryos not expressing *Dfd-EYFP* were selected and analyzed.

### Screen Strategy

On the basis of Rein's work [3] and our own experiments, a sensitized screen was conducted by crossing deficient second chromosome stocks with *Df(3L)DocA* flies. Each deficiency stock was crossed to the strain that carried an embryonic marker in the second chromosome balancer. Progeny with both the heterozygous mutant allele and embryonic marker were collected and crossed to deficient *DocA*, with *Dfd-EYFP* in the balancer, and *Toll-GFP* on X chromosome. Embryos from the second cross were checked under the fluorescence microscope. Double heterozygous embryos with both the deleted region and deleted *Doc* genes were separated by their lack of *Dfd-EYFP*. Embryos were gathered and screened 6–10 times, totally approximately 200 embryos checked per cross.

The Chi-square test indicated that if 20 mutant hearts out of a total of 200 embryos was indicative of a statistically significant difference between double heterozygous embryos and single heterozygous embryos (p<0.1). This percentage was derived from a control using wild-type heterozygous *Df(3L)DocA* embryos where there were 14 mutant hearts in 263 embryos. The final step of the screen was to narrow down the relevant regions which had statistically good mutant percentages, in order to find genes within those regions that can function in coordination with *Doc*. Embryos that were homozygous for the mutant allele were assessed for dorsal vessel phenotypes in the following ways: (1) evaluation of cardioblast assembly during the development of the dorsal vessel; (2) verification that the cardioblasts were properly ordered into two parallel rows of adjoining cells; (3) confirmation that each of the 104 cardioblasts normally present in a phenotypically wild-type dorsal vessel was present and properly specified; and finally (4) assessment of the ability of the cardioblast rows to migrate along the ectoderm as the dorsal vessel closes.

### Immunohistochemistry

Various single- and double-label immunohistochemistry analyses were performed as previously described [18]. The following primary antibodies were used in these studies: mouse anti-β-galactosidase, 1∶300 (Promega, Madison, WI); rabbit anti-Mef2, 1∶1,000 (H. Nguyen); mouse anti-α-Spectrin, 1∶200 (Developmental Studies Hybridoma Bank); rabbit anti-Tin, 1∶750; rabbit anti-Doc2, 1∶2000 and rabbit anti-Pnr, 1∶3000 (M. Frasch) [3]; rabbit anti-Eve, a 1∶2000 dilution (J. Skeath); rabbit anti-Spir KIND, 1∶200 (E. Kerkhoff). A mouse anti-SpirD antibody (1∶100 dilution) was used to detect the D-isoform or N-terminus of the A-isoform, while a mouse anti-SpirC3 antibody (1∶100 dilution) was used to detect the C-isoform or the C-terminus of the A-isoform (S.M. Parkhurst) [16]. These Spir antibodies were used to determine the localization of endogenous Spir in wild-type *Drosophila* embryos through immunofluorescence staining. Primary antibodies were detected with Alexa Flour 488 goat anti-rabbit and goat anti-mouse (1∶400), and Alexa Fluor 555 goat anti-rabbit (1∶400) (Invitrogen Molecular Probes, Carlsbad, CA). Images were obtained with a Zeiss Axioplan 2 microscope and a Nikon A1R laser scanning confocal microscope.
